# Impact of same day screening mammogram results on women’s satisfaction and overall breast cancer screening experience: a quality improvement survey analysis

**DOI:** 10.1186/s12905-022-01919-3

**Published:** 2022-08-08

**Authors:** Biren A. Shah, Anicia Mirchandani, Srishti Abrol

**Affiliations:** 1 Department of Diagnostic Radiology, Detroit Medical Center, Wayne State University, Detroit, MI USA; 2 Envision Healthcare, Nashville, USA; 3 Department of Radiology, Detroit Medical Center, Sinai-Grace Hospital, 6071 West Outer Drive, Detroit, MI 48235 USA

**Keywords:** Mammogram, Breast imaging, Diagnostic radiology, Mammogram results

## Abstract

**Background:**

Most women undergoing screening examinations in the U.S. do not receive immediate results and for many this results in increased stress, inconvenience, delayed diagnosis, and potential loss to follow-up.

**Objective:**

To study the impact of same appointment mammogram results on breast cancer screening experience and patient satisfaction.

**Materials and methods:**

A 6-question survey with questions focused on breast cancer screening experience with our new service of same appointment mammogram results was distributed to 200 patients, with 185 patients returning their responses. Patients evaluated their current experience on receiving their screening results during the same appointment with their prior breast cancer screening experience. Patients who did not respond to their satisfaction score either before or after same appointment results were excluded from the patient cohort analyzing satisfaction score. Remaining questions were analyzed separately as additional satisfaction assessment tools.

**Results:**

About 48% of the patients indicated an improvement in their screening experience with same appointment mammography results service, while 47% of the patients reported no significant difference in their experience.

**Conclusion:**

Although not statistically significant, same appointment mammogram results were able to make a positive impact on breast cancer screening experience among 48% of the patients. Further research elucidating barriers to screening and other ways to improve patient satisfaction will be required to increase breast cancer screening compliance.

**Supplementary Information:**

The online version contains supplementary material available at 10.1186/s12905-022-01919-3.

## Introduction

Breast cancer is the most common non-skin cancer for women in the United States [1]. The most recent data from the CDC found that 67% of women in the US had a mammogram with the past two years [2]. Despite the widespread implementation of screening mammography, breast cancer screening is associated with significant anxiety and psychological distress [3]. Previous studies have shown that exam compliance is related to patient experience. In a study done by Glockner et al., “personal experience” was one of the highest incentives toward getting screening mammography done [4]. In another study by Giersch et al., part of the 54% of women who did not maintain screening adherence over three years cited decreased satisfaction with their mammography experience as one of the major contributing factors [5].

The benefits of breast cancer screening, however, have consistently been demonstrated. Mortality related to breast cancer has steadily decreased in women over 50 in the past 25 years. In a metanalysis by Dibden et al. reviewing 27 published studies on breast cancer screening programs and trials worldwide, about 22% reduction in mortality was reported with invitation to screening and 33% reduction in mortality with actual screening [6].

Although there are multiple imaging modalities such as mammography, ultrasonography and MRI, mammography remains the primary modality for initial breast cancer screening and is the only modality which has been demonstrated to reduce mortality. New guidelines by American College of Radiology (ACR) and Society of Breast Imaging (SBI) recommend annual breast cancer screening for women in the US at average risk starting at age 40, with evaluation of all women for high breast cancer risk starting at age 30 [7, 8]. Despite various health organizations advocating the necessity of breast cancer screening and increased awareness among the patient population, there are multiple barriers affecting screening rates. The screening rates have been linked to fear of detection of cancer, lack of motivation and anxiety in addition to socioeconomic factors [9, 10]. A study by Bull et al. analyzing questionnaires filled by women at different stages of breast cancer screening from screening invitation, recall and proceeding to biopsy reported that at least 10% of the screened women suffered anxiety due to fear of having breast cancer, and about 10% of women who proceeded to undergo biopsy required psychological therapy and counselling. Multiple studies have raised concerns on screening related anxiety among women due to delay in test results stressing upon the need for expeditious and reliable screening services [11, 12].

In an attempt to reduce breast cancer screening related anxiety and test result wait times, our urban academic institution decided to provide the option for screening mammogram to referred patients who request same day results at the time of check-in. By same day results, this meant that patients would receive their screening results and that additional diagnostic imaging would be performed on the same day of service.

Those patients that requested same day mammogram, were also asked to participate in a voluntary anonymous quality improvement survey to assess the impact it has on their screening experience.

## Materials and methods

### IRB approval

This study was conducted in compliance with HIPAA guidelines and was deemed exempt by the IRB review board after a detailed review.

### Patient cohort

A six-question anonymous survey was given to 200 patients from June 2020—November 2020 who opted for same day screening mammogram results at the time of check-in. 185 of those 200 patients completed the given survey.

### For same day screening results

After completion of the mammogram, this would be read by the breast radiologist with the use of computer aided detection artificial intelligence. The technologist would then inform the breast radiologist, that the patient was waiting to receive their results. After reviewing the mammogram, the radiologist would speak to the patient in a private room to convey the results. Patients were also informed that their ordering provider would receive the mammogram report and that they can expect to receive a letter in the mail that reiterates in writing the results of their screening mammogram. After relaying the results to the patient, the patients were then asked by the radiologist whether they would be willing to complete an anonymous six-question survey regarding their experience and to then drop the survey into a locked box in the patient dressing area.

The six-question survey focused on different aspects of patient satisfaction reflecting in their responses such as average wait times following the mammogram to receive screening results, patient satisfaction with and without same day appointment result, willingness to return to our facility for their next screening mammogram, willingness to recommend our facility to their family and friends, and if they would prefer to receive results over phone at a later time than in person during the same appointment. (See Additional file 1 of the survey questions.)

## Results

Of the 200 surveys distributed, 185 patients completed the survey. These 185 patients acted as their own controls as they were asked to compare their satisfaction level with their prior appointments when they did not receive same appointment results. Some patients chose not to answer some of the questions and those patients were excluded from analysis of each question individually.

One hundred fifty four patients out of the 185 patients (83.2%) indicated they received their screening results in under 30 min during the same appointment. one hundred seventy nine patients (96.7%) wanted to return to our facility for their next screening appointment and would recommend our facility to their friends and family. 153 (82.7%) responded in favor of same appointment results rather than long wait times and delayed results over the phone.

Thirty patients did not provide a response to either question rating their satisfaction level with and without same appointment results due to no prior screenings to assess for the difference and were thus excluded from analysis. Therefore, we evaluated responses from the remaining 155 patients. These two questions were rated on a scale of 0–10 which provided us with a variety of responses. The satisfaction score from their prior experience was subtracted from their same day appointment satisfaction score to determine the net change in satisfaction. An improvement in screening experience was defined as a positive score: 1 or greater. No change in screening experience was defined as a score of 0. Decreased satisfaction of screening experience was defined as a negative score.

**Fig. 1:**
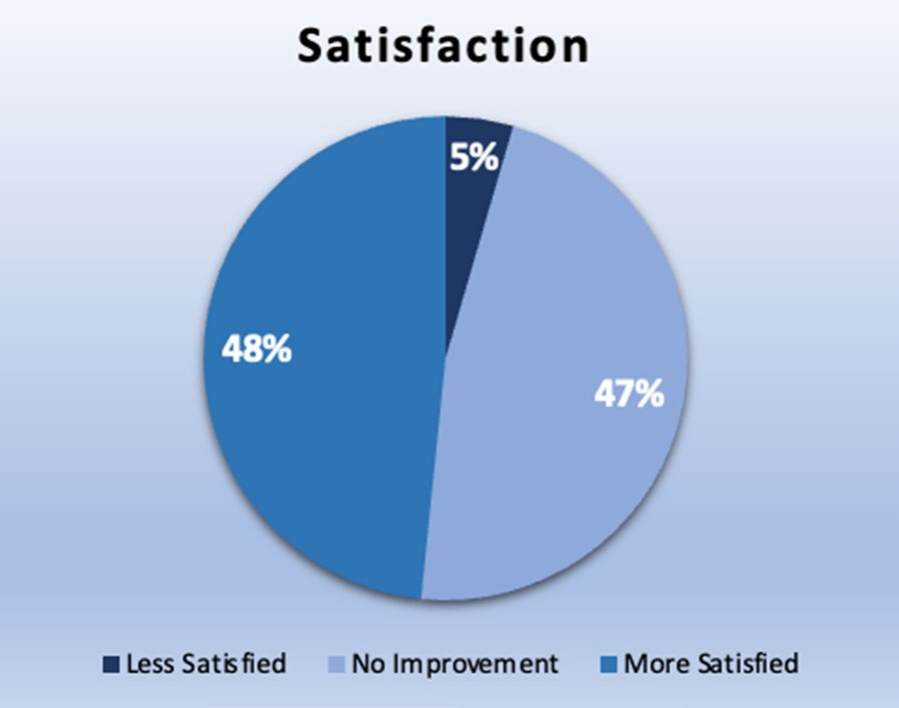
Pie chart illustrating distribution of patients based on their satisfaction level after receiving same appointment screening results when compared to their old screening appointment without same day results

**Fig. 2:**
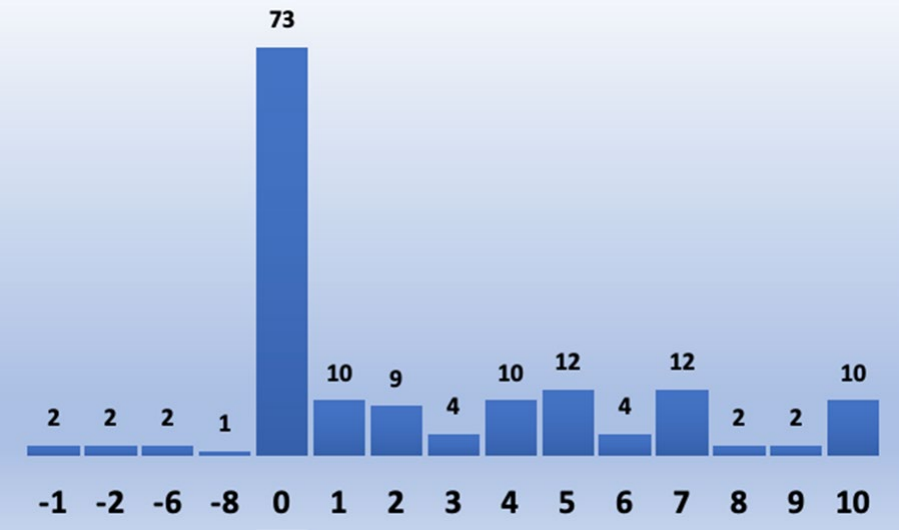
Histogram illustrating difference in points ranging 010 estimating the change in screening experience after same appointment screening results. 0 indicates no difference in satisfaction score, below 0 indicates negative difference in satisfaction score and above 0 indicates positive difference in satisfaction score

73 (47%) of the patients reported no difference in their screening experience, with a score of 0. 75 (48%) patients reported improvement in their screening experience, with a score of 1 or greater. Although not statistically significant (*p*-value ≤ 0.05), 48% patients reported significantly improved screening experience. It should also be noted that 7 (5%) out of 155 patients reported a worse experience with same appointment screening results. (Figs. 1 and 2).

## Discussions

Our study showed a positive impact of reduced wait times in at least 48% of patients who demonstrated improvement in their screening experience with same day appointment results but this was not shown to be statistically significant. Additionally, 47% patients showed no difference in their experience. About 5% of the patients were less satisfied with the same appointment results, which may be related to multiple factors such as initial patient expectations, longer than expected wait times, lack of understanding of clinic workflow and feeling pressured into waiting for result during the same appointment with not understanding their choice to reject the proposal. Of the 185 patients who filled our survey, over 154 (83.2%) patients received their results in under 30 min after the mammogram and majority of the remaining patients received results in under 45 min which is conceivable in our clinic workflow experience. This is significantly quicker than delivery of screening mammogram results which is typically on the order of days. However, 4 patients (2.2%) reported waiting time longer than an hour which may be due to independent patient factors considering wait time starting from the time to check-in, getting the screening mammogram exam, to ultimately waiting for the results. Other factors such as needing to obtain outside films or obtaining additional views was considered, but was not found to play a role in the increased wait times for these patients.

Willingness to return to our breast imaging center next year for a screening mammogram acted as an important assessment tool indicating that our service of same appointment results was well received by our patients with 96.7% of the patients wanting to return for future screening for same appointment results. It was also remarkable that 96.7% responded in favor of recommending our same appointment results service to their family and friends. Additionally, only 31 (16.7%) patients indicated their preference of being notified of their results over phone as opposed to 153 (82.7%) patients showing preference to receiving results in person than over phone. Thus, in our experience patients preferred results in person over results via phone or mail, making screening a more personable and pleasant experience for our patients. In a previous study by our group, it was evident that patients preferred results either during the same appointment or within 48 h of the screening appointment through a follow up appointment or over the telephone being the most desirable methods [13].

Breast cancer screening can be a stressful experience for women in having to wait several days or even weeks to know the results of their screening mammogram. Sometimes, a screening mammogram result may require a recall visit for additional imaging that could take days or weeks until the patient can get their diagnostic work-up. The anxiety and fear, compounded with the uncertainty and prolonged wait times, makes screening mammography an unpleasant experience which affects patient compliance rates [14–16]. Multiple factors can further delay the breast cancer screening in different settings such as physician recommendation, lack of same-day mammography availability, lack of weekend and evening appointments for working women, lower mammography capacity of the clinics, limited notification methods, with majority of patients receiving results via mail within 30 days as per Mammography Quality Standards Act (MQSA) guidelines [17, 18].

In a controlled trial previously done which studied the impact the availability of same day screening mammography had on patients, it was reported that same-day mammography effectively increased the adherence to breast cancer screening recommendations among women aged 50 years or older and improved patient satisfaction [19]. Thus, it was again demonstrated that factors reducing the diagnostic interval have often been associated with better patient satisfaction rates in all cancer screening, reducing psychological distress and anxiety among the patients.

The probability of a patient returning for rescreening after a negative mammogram is directly related to their initial screening experience with increasing number of women avoiding rescreening if they feel dissatisfied with the service provided by the staff, longer wait times, inability to schedule appointments at a convenient time and embarrassment going through the screening [20]. Breast cancer is now the most commonly diagnosed cancer as per 2020 cancer statistics and regular screening is essential to detect it in early stages to reduce mortality. It can be achieved with simple steps that make breast cancer screening a less cumbersome and a patient-oriented personalized experience.

While this study showed that 48% of patient had improvement in their screening experience with same day appointment results, there were still limitations to our study. First, patients were expected to rate their satisfaction of screening experience compared to prior appointments, which could have led to recall bias. This could have been mitigated by having a control group vs those who were randomized to partake in same day appointment results. Additionally, not having a randomized control group could have subject our study to selection bias as to who voluntarily participated in same day appointment results. Also, no sociodemographic information was collected on these participants. This could potentially be a confounder in our results and could be an area for further research. Lastly, our study was conducted at a single center which may make so that our data is not necessarily representative of other patient populations.

## Conclusion

Breast Cancer screening is associated with anxiety and distress which in turn has been shown to be related to reduced compliance in breast cancer screening. Our study sought to demonstrate whether same day appointment results could increase patient satisfaction of breast cancer screening. While a large proportion of participants reported increased satisfaction, this was not found to be statistically significant but may still have clinical utility. Further multi-institutional studies and controlled trials may highlight the benefit that same day appointment results can have on increasing patient satisfaction by decreasing the time that elapses between screening and notification of mammography results.

## Supplementary Information

**Additional file 1.** Survey enlisting all questions that were used to conduct this quality improvement study.

## Data Availability

The authors declare that they had full access to all of the data in this study and the authors take complete responsibility for the integrity of the data and the accuracy of the data analysis.
