# Supplementary figures and images for: Impact of same day screening mammogram results on women’s satisfaction and overall breast cancer screening experience: a quality improvement survey analysis
Source: BMC Womens Health. 2022 Aug 8;22:338. doi: 10.1186/s12905-022-01919-3 (PMC9361536; doi:10.1186/s12905-022-01919-3)

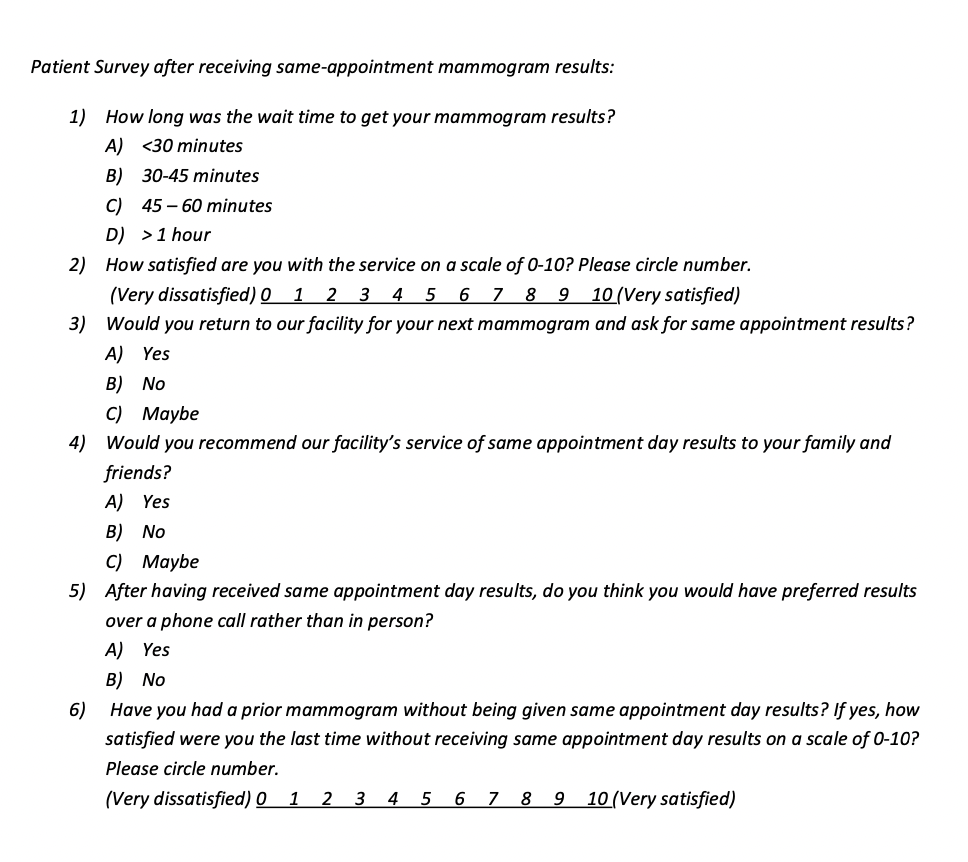


Survey enlisting all questions that were used to conduct this quality improvement study.

Supplement: Supplementary file 1 — Additional file 1. Survey enlisting all questions that were used to conduct this quality improvement study. [file 12905_2022_1919_MOESM1_ESM.docx]
